# Activation of the Human Angiotensin-(1-12)-Chymase Pathway in Rats With Human Angiotensinogen Gene Transcripts

**DOI:** 10.3389/fcvm.2019.00163

**Published:** 2019-11-15

**Authors:** Carlos M. Ferrario, Jessica VonCannon, Sarfaraz Ahmad, Kendra N. Wright, Drew J. Roberts, Hao Wang, Tomohisa Yamashita, Leanne Groban, Che Ping Cheng, James F. Collawn, Louis J. Dell'Italia, Jasmina Varagic

**Affiliations:** ^1^Department of Surgery, Wake Forest School of Medicine, Winston-Salem, NC, United States; ^2^Department of Social Science and Health Policy, Wake Forest School of Medicine, Winston-Salem, NC, United States; ^3^Department of Physiology-Pharmacology, Wake Forest School of Medicine, Winston-Salem, NC, United States; ^4^Department of Anesthesia, Wake Forest School of Medicine, Winston-Salem, NC, United States; ^5^Section on Cardiovascular Center, Wake Forest School of Medicine, Winston-Salem, NC, United States; ^6^Department of Cell, Developmental, and Integrative Biology, University of Alabama at Birmingham School of Medicine, Birmingham, AL, United States; ^7^Division of Cardiovascular Disease, University of Alabama at Birmingham School of Medicine, Birmingham, AL, United States

**Keywords:** chymase, cardiac hypertrophy, hypertension, renin angiotensin system, transgenic rats

## Abstract

Angiotensin-(1-12) [Ang-(1-12)], an alternate substrate for tissue angiotensin II (Ang II) formation, underscores the importance of alternative renin-independent pathway(s) for the generation of angiotensins. Since renin enzymatic activity is species-specific, a transgenic model of hypertension due to insertion of the human angiotensinogen (AGT) gene in Sprague Dawley rats allowed for characterizing the contribution of a non-renin dependent mechanism for Ang II actions in their blood and heart tissue. With this in mind, we investigated whether TGR(hAGT)L1623 transgenic rats express the human sequence of Ang-(1-12) before and following a 2-week oral therapy with the type I Ang II receptor (AT1-R) antagonist valsartan. Plasma and cardiac expression of angiotensins, plasma renin activity, cardiac angiotensinogen, and chymase protein and the enzymatic activities of chymase, angiotensin converting enzyme (ACE) and ACE2 were determined in TGR(hAGT)L1623 rats given vehicle or valsartan. The antihypertensive effect of valsartan after 14-day treatment was associated with reduced left ventricular wall thickness and augmented plasma concentrations of angiotensin I (Ang I) and Ang II; rat and human concentrations of angiotensinogen or Ang-(1-12) did not change. On the other hand, AT_1_-R blockade produced a 55% rise in left ventricular content of human Ang-(1-12) concentration and no changes in rat cardiac Ang-(1-12) levels. Mass-Spectroscopy analysis of left ventricular Ang II content confirmed a >4-fold increase in cardiac Ang II content in transgenic rats given vehicle; a tendency for decreased cardiac Ang II content following valsartan treatment did not achieve statistical significance. Cardiac chymase and ACE2 activities, significantly higher than ACE activity in TGR(hAGT)L1623 rats, were not altered by blockade of AT_1_-R. We conclude that this humanized model of angiotensinogen-dependent hypertension expresses the human sequence of Ang-(1-12) in plasma and cardiac tissue and responds to blockade of AT_1_-R with further increases in the human form of cardiac Ang-(1-12). Since rat renin has no hydrolytic activity on human angiotensinogen, the study confirms and expands knowledge of the importance of renin-independent mechanisms as a source for Ang II pathological actions.

## Introduction

The downstream cascade of biologically active angiotensins is initiated from the hydrolysis of angiotensinogen (AGT), a 485 amino acid protein of primary hepatic origin. While renal renin is recognized as the primary enzyme accounting for the cleavage of the N-terminus of AGT at the 10^th^ amino acid position of the molecule (1), there is limited awareness that the renin cleavage of AGT leading to angiotensin I (Ang I) formation exhibits significant species-specificity due to the presence of amino acid differences between the 10^th^ and 11^th^ amino acids of the protein. Whereas mice and rat AGT sequence contains leucine at position 10^th^ and 11^th^ of the C-terminus, valine rather than leucine is present at the 11^th^ position of human AGT (hAGT) (1). Differences in the molecular sequence of AGT between rodents and humans impairs the enzymatic activity of rodent renin to digest the human AGT protein (2, 3).

Renin is not the only enzyme cleaving the angiotensin II (Ang II) generating substrate. The lysosome serine proteases cathepsin G, D, and E possess Ang I-forming activity while tonin, a serine cytosolic enzyme, forms Ang II directly from AGT (4). New studies underscore a need to reexamine the existence of non-renin dependent mechanisms for angiotensin peptide formation through the detection of intermediate substrates that are cleaved from circulating and tissue AGT. An AGT-derived N-terminal sequence, named angiotensin-(1-12) [Ang-(1-12)] (5) is present in the blood and tissues of normotensive (5, 6) and hypertensive rats (7–9) and in the human heart (10, 11).

In exploring Ang-(1-12)'s role as an endogenous substrate for direct Ang II formation, we characterized a humanized model of hypertension associated with cardiac hypertrophy and systolic dysfunction (9). These transgenic rats were created by Ganten and collaborators (2) to establish a colony of rats with genomic insertion of both the hAGT and human renin genes (double transgenic rats) (12, 13). Transgenic rats [TGR(hAGT)L1623] exhibit sustained hypertension, cardiac hypertrophy, associated with significant increases in cardiac Ang-(1-12) immunofluorescence and a 4-fold increase in cardiac Ang II content (9). Since endogenous rat renin does not cleave hAGT (2), this humanized model of hypertension provides a unique opportunity to characterize non-renin dependent mechanisms for systemic and cardiac Ang II generation through endogenous Ang-(1-12) processing. Given that the C-terminal amino acids of the rat and human dodecapeptide do not share the same amino acid sequence, the study described here required the generation of antibodies with separate binding epitopes for the human Ang-(1-12) and rat forms of the peptide. The present study investigated whether TGR(hAGT)L1623 transgenic rats express the human sequence of Ang-(1-12) in their blood and heart in the absence and in the presence of sustained blockade of Ang II by a 2-week oral therapy with the type I Ang II receptor (AT_1_-R) antagonist valsartan.

## Materials and Methods

### Study Protocol

This study was performed in 22 male adult rats of the Sprague Dawley (SD) strain genetically engineered to express the human sequence of the AGT gene. Eleven of these rats were instrumented at age 10 weeks with implantable telemetry transducers for continuous measures of arterial pressure and heart rates. Surgical procedures were performed as detailed by us elsewhere (9, 14). A second group of TGR(hAGT)L1623 rats (*n* = 11), not receiving telemetry probes facilitated a more precise characterization of renin angiotensin system components in blood and heart tissue. Four other adult male SD rats were added to the study for the purpose of assessing control content of Ang II in the heart by Mass Spectroscopy. All rats were housed in pairs in a facility on a 12-h light/dark cycle with *ad libitum* access to rat chow (Teklad Global 16% Protein Rodent Diet, Envigo, Madison, WI) and tap water. Procedures were carried out in accordance with National Institutes of Health Guide for the Care and Use of Laboratory Animals and approved by the Institutional Animal Care and Use Committee of Wake Forest University Health Sciences.

All twenty-two TGR(hAGT)L1623 rats were randomized to drink either vehicle (Veh) or the Ang II receptor antagonist,—valsartan (30 mg/kg/day),—for two weeks. Monitoring the amount of water intake every 24 h allowed medication dose-adjustments every third day. At the completion of the study, rats were euthanized by decapitation and trunk blood, tibia, heart, and kidney tissues were rapidly removed and fractioned in small pieces. Tissue samples were frozen in liquid nitrogen or dry ice for later measures of angiotensins and Ang II-forming enzyme activities. Removed organs were weighed and tibia length was recorded using a micrometer.

### Physiological Measures

Continuous monitoring of arterial pressure waveforms with concomitant registration of beat-by-beat heart rate and motor activity were obtained in TGR(hAGT)L1623 rats via chronically implanted telemetry probes throughout a time that commenced two weeks after transducer's implantation and continued during the two weeks in which rats drank tap water containing either the vehicle (*n* = 5) or valsartan (10 mg/kg/day; *n* = 6). The electronic output of the implanted telemetry probes was analyzed by the provided specialized software (Data Science International, St. Paul, MN).

Transthoracic echocardiography (VevoLAZR Imaging system; VisualSonics, Toronto, Canada) was performed at week 2 following vehicle or valsartan therapy as described elsewhere (15). Left Ventricular (LV) M-mode images allowed determination of posterior wall thickness (PWT), interventricular septal wall thicknesses (IVS) at end-diastole, and LV end-diastolic and end-systolic dimensions (ESD and EDD, respectively). Additional variables included assessment of stroke volume, cardiac output, ejection fraction (EF), and fractional shortening [FS (%). Mitral valve early filling velocities (E) and septal annular velocities (e') were obtained using a pulsed Doppler and tissue Doppler, respectively. E/e' was calculated as a measurement of LV filling pressure.

### Biochemical Procedures

#### Plasma Renin Concentration, Plasma, and Cardiac Angiotensins Assays

Plasma renin concentration (PRC), plasma and left ventricular tissue rat Ang-(1-12) [rAng-(1-12)] and hAng-(1-12), Ang I and Ang II were measured by radioimmunoassays (RIA) as described previously (9). In addition, left ventricular tissue was submitted to Attoquant Diagnostics GmbH (Vienna, Austria) for measurements of cardiac tissue angiotensins using Liquid Chromatography with tandem mass spectrometry (LC/MS-MS) (16, 17). Serum hAGT concentrations (ELISA) and cardiac AGT protein (Western Blot) were determined as described elsewhere (9).

A new RIA procedure for measurement of hAng-(1-12) in plasma and heart tissue was incorporated in this study using a polyclonal antibody directed toward the C-terminus of the hAng-(1-12) sequence. Briefly, blood was rapidly collected in pre-chilled tubes containing a cocktail of inhibitors [1,10-ortho-phenanthroline (0.5 mM); p-hydroxymercuribenzoate (1 mM); pepstatin A (125 μM); EDTA = 5 mM]. Plasma (500 μL) was diluted to 3 mL volume with 0.1% HFBA and processed for Ang-(1-12) content. Heart tissue (~50–60 mg) was boiled for 5 min in 3 mL of 1% n-heptafluorobutyric acid (HFBA), homogenized and centrifuged at 28,000 × g for 10 min at 4°C. Angiotensin peptide fragments were concentrated by passing the supernatant through activated C18 solid phase extraction (SPE) columns. Peptides were eluted from the SPE column with 3 mL of methanol:HFBA mixtures (80%:0.1%) and eluted samples were placed in a SpeedVac to evaporate the solvent. The dried samples were reconstituted with 0.1 mL of RIA buffer containing 50 mM phosphate buffer saline, 25 mM EDTA, 0.5% triton X-100, 0.05% sodium azide and 0.5% BSA (pH 7.4). Next, highly purified radiolabeled ^125^I-Ang-(1-12) human sequence [purity ≥ 98%; ~25,000 cpm/RIA tube] and 50 μL of affinity purified human anti-Ang-(1-12) antibody (1:400) were added and incubated for 18–20 h at 4°C. At the end of incubation period, 50 μL of 1% γ-globulin was added, mixed and the bound/free [B/Bo] forms of hAng-(1-12) were then separated by precipitating the bound fraction with 200 μL of 23% polyethylene glycol (PEG). The radioactivity corresponding to the antibody-bound antigen in the precipitate was measured in a Packard Cobra II Auto-Gamma Counter. The amount of hAng-(1-12) in the tissue and plasma extracts were estimated from a calibration curve for synthetic hAng-(1-12) standard ranging from 0 to 150 ng/RIA tube. The minimal detection limit of the hAng-(1-12) peptide was 0.3 ng/RIA tube (1.5 ng/mL).

#### Cardiac Angiotensin Converting Enzyme (ACE), ACE2, Chymase mRNAs, and Enzymatic Activities

Total RNA was isolated from the heart using the RNeasy Lipit Tissue Mini Kit (Qiagen, Inc., Germantown, MD) and further purified using RNeasy MinElute Cleanup Kit (Qiagen, Inc., Germantown, MD) followed by quality assessment on an Agilent 2100 bioanalyzer, as described previously (18, 19). Relative quantification of mRNA levels by real-time qPCR for ACE, angiotensin converting enzyme 2 (ACE2), mast cell proteases (MCP-1, and MCP-5) was performed using a SYBR Green PCR kit (Qiagen, Inc., Germantown, MD) (19). Amplification and detection were performed with the QuantStudio 3 real-time PCR Systems (Applied Biosystems, Foster City, CA). Sequence-specific oligonucleotide primers were designed according to published GenBank sequences and confirmed with OligoAnalyzer 3.0. The relative target mRNA levels in each sample were normalized to GAPDH. Expression levels are reported relative to the mean value of the control group.

Cardiac ACE and ACE2 activities were determined, as described elsewhere (20) in plasma membranes obtained from the hearts of TGR(hAGT)L1623 rats using ^125^I-Ang I and ^125^I-Ang II, respectively. The method described by Ahmad et al. (20) was employed for determination of chymase enzymatic activities with radiolabeled hAng-(1-12) as the substrate.

#### Western Blot Analysis of Left Ventricular Angiotensinogen and Chymase

Frozen heart tissue from TGR(hAGT)L1623 transgenic rats treated with vehicle or valsartan was homogenized in a buffer containing 10 mM HEPES (pH 7.4), 125 mM NaCl, 1 mM EDTA, 1 mM NaF, 10 μg/ml leupeptin, 10 μg/ml pepstatin A, and 1 mM PMSF final concentrations. Homogenates were centrifuged at 2,000 g, and then at 100,000 g for 60 min at 4°C. Protein concentration in supernatant fractions was determined by the Bradford method using a BioRad kit (Bio-Rad, Hercules, CA). Samples of supernatant were separated by gel electrophoresis, and then proteins were eluted from the gels to Hybond PVDF membranes (Bio-Rad, Hercules CA) for 1 h at 100 V. Nonspecific binding was blocked in 5% nonfat dried milk in 0.1% Tween 20 in TBS for 60 min at room temperature. The blots were incubated with either an anti-rat chymase antibody (1:1000, Cloud-Clone Corp., Katy, TX), an anti-human AGT (1:5000; R&D Systems, Minneapolis, MI) or anti-rat AGT antibody (1:1000, gift from Dr. Chappell MC) raised against human (residues 34–485) and rat angiotensinogen (residues 44–56), respectively. The immunoblots were then resolved with Pierce Super Signal West Pico Chemiluminescent substrates as described by the manufacturer and imaged using a Bio-Rad Chemidoc MP imager. For loading control, TGX Stain-Free™ Precast gels (Bio-Rad) were used and activated prior to transfer to obtain total protein load using the Bio-Rad Chemidoc MP imager. Signal quantification was performed using an image analysis program (Image Lab, Bio-Rad, Hercules, CA) and optical densities were expressed as the ratio between corresponding protein and total protein.

#### Chymase Cardiac Immunofluorescent Imaging

Whole heart tissue samples from SD and TGR(hAGT)L1623 hypertensive rats were prepared as formalin-fixed paraffin embedded (FFPE) tissues, and sections placed and dried on glass slides for later processing. The glass slides were heated for 30 min at 55°C on a slide warmer and then placed into slide holders and re-hydrated/de-waxed through a series of immersions following Xylene, and graded dilution of ethylene-oxide (from 100 to 70%) in PBS. The slides were then immersed in the Antigen Unmasking Solution (Vector Laboratories, Burlingame CA) and placed in a 900 watt microwave oven on high for 3 min 30 sec, cooled for 1–2 min and fluid level replenished as needed, microwaved again, and allowed to cool to room temperature for 20 min. The slides were then rinsed in PBS. Slides were removed from the PBS and blocked with a solution of 1% bovine serum and 5% normal goat serum in PBS for 1 h at room temperature. With slides facing away, blocking solution was washed away with DDH20 being careful not to disturb the tissue. The slides were then placed face up and each section surrounded with a circle from an ImmEdge pen (Vector Laboratories, Burlingame CA) to create a hydrophobic barrier around the tissue. To stain for membrane proteins, 500 μL of a 1:200 Wheat Germ Agglutinin AlexaFluor 488 conjugate in blocking solution (Life Technologies, Eugene OR) was added to each tissue section in the dark for 10 min, and then washed with PBS. All steps from this point forward were performed in the dark. To detect the primary antigen, we then added 500 μL of a 1:100 rabbit α-chymase antibody in blocking solution (CloudClone, Katy TX) overnight in a humidified chamber at 40°C. The following day, the slides were rinsed with DDH20, and then PBS, blocked once more with 1% bovine serum and 5% normal goat serum in PBS for 30 min at room temperature. For fluorescent detection, we then added 500 μL of a 1:800 goat α-rabbit IgG highly cross absorbed AlexaFluor 568 antibody (Thermo Fisher, Waltham MA) in blocking solution for 1 h at room temperature, and then rinsed with PBS. The slides were blotted to remove as much moisture as possible without disturbing the tissue, and then cover slipped with Vectashield Hard Set Mounting Medium containing DAPI (Vector Laboratories, Burlingame CA). Slides were sealed with clear nail polish and allowed to dry overnight at 40°C. Slides were imaged on an Olympus FV1200 Laser Scanning Confocal Microscope (IX83 inverted platform) with 60x oil immersion lenses. Images were analyzed with Olympus FluoView FV10-ASW Version 04.02.

### Statistics

Data are expressed as means ± standard error of the mean (SEM) unless expressed otherwise. Analysis of differences was assessed by one-way ANOVA using Prism software (GraphPad Version 7.0, San Diego, CA). The Pearson correlation coefficient (*r*) was employed to evaluate the linear correlation between two variables that were demonstrated to be normally distributed. A positive or negative value of *r* between 0.70 was set as the cut-off for defining the strength of the association between two variables. Statistical significance was set at *p* < 0.05.

## Results

Mean 24 h arterial pressure (± SD) during the 5 days preceding the intervention averaged 146 ± 5/101 ± 3 mm Hg in vehicle-treated rats (*n* = 5) and 143 ± 4/97 ± 3 mm Hg in the six rats randomized to receive the AT_1_ receptor antagonist valsartan (*p* > 0.05). Corresponding mean 24 h heart rates (± SD) averaged 354 ± 15 beats/min in the vehicle treatment group and 354 ± 13 beats/min in rats destined to be medicated with valsartan. The treatment had no effect in body weights. Valsartan treatment was associated with a statistically significant decrease in left ventricular PWT (Table 1).

**Table 1:** Echocardiographic measures in vehicle and valsartan treated TGR(hAGT)L1623 rats.

**Variable**	**Vehicle treated group**	**Valsartan-treated group**	***P*-value**
Heart rate (beats/min)	372 ± 7	367 ± 6	n.s.
Stroke volume (μl)	268 ± 10	285 ± 17	n.s.
Cardiac output (mL/min)	99 ± 4	105 ± 6	n.s.
Ejection fraction (%)	68 ± 2	70 ± 1	n.s.
Fractional shortening (%)	40 ± 1	42 ± 1	n.s.
Left ventricular end diastolic diameter (mm)	8.47 ± 0.17	8.59 ± 0.24	n.s.
Interventricular septal wall thickness (mm)	1.95 ± 0.05	1.82 ± 0.09	n.s.
Left ventricular posterior wall thickness (mm)	2.43 ± 0.07	2.14 ± 0.06	0.0068
Isovolumic relaxation time (ms)	23 ± 0.8	22 ±0.5	n.s.
Mitral valve early filling velocity (mm/s)	985 ± 21	991 ± 21	n.s.
Mitral valve annular velocity (mm/s)	72 ± 2	74 ± 2	n.s.
E/e' ratio	14 ± 0.6	13 ± 0.4	n.s.

*Values are means ± standard error (SE) of the mean (n = 11 for vehicle control and n = 11 for rats medicated with valsartan)*.

**Figure 1:**
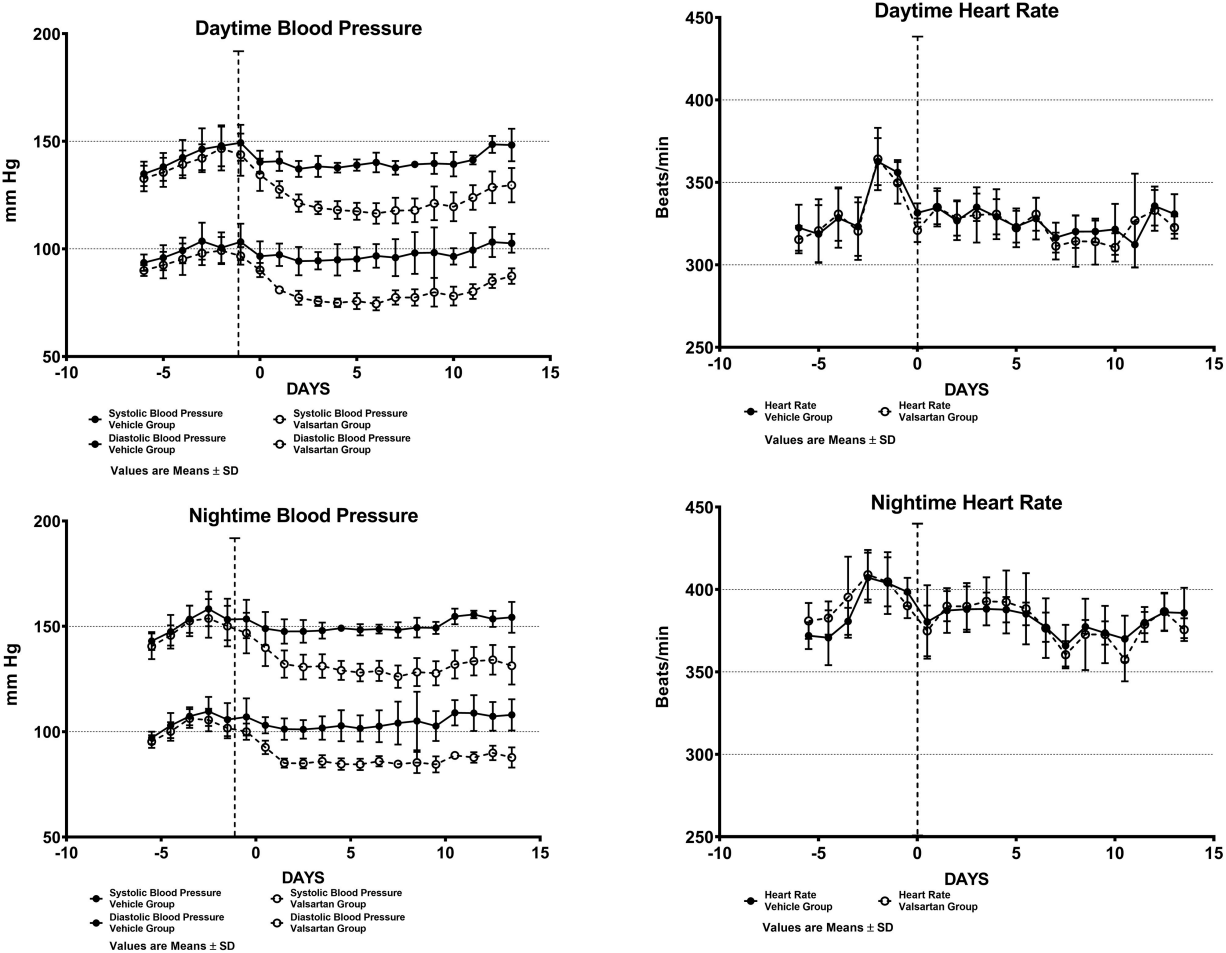
Arterial blood pressure and heart rate measures from chronically implanted radiotelemetry probes in transgenic rats expressing the human sequence of the angiotensinogen gene before and throughout a fortnight exposure to oral therapy with the AT_1_-R antagonist valsartan. Values are means ± SD of daytime (upper panels) and nighttime (lower panels) of systolic and diastolic blood pressure daily averages.

Figure 1 shows daytime and nighttime arterial pressure and heart rate values of the two groups before and during 2 weeks of exposure to either vehicle (CONTROL GROUP) or valsartan (TREATMENT GROUP). As reported elsewhere (9), the nocturnal exploratory and feeding behaviors in transgenic rats expressing the human AGT gene is associated with higher values of both arterial pressure and heart rate (Figure 1). *Nighttime* averages of mean arterial pressure and heart rate are 126 ± 5 (± SD) mm Hg and 389 ± 16 (± SD) beats/min in the CONTROL GROUP and 123 ± 4 (± SD) mm Hg and 394 ± 11 beats/min (± SD) in rats assigned to the TREATMENT GROUP. Corresponding 5-day baseline averages for *daytime* mean arterial pressure in the CONTROL and TREATMENT groups are 119 ± 5 (± SD) mm Hg and 115 ± 4 (± SD) mm Hg, respectively. *Daytime* baseline heart rates averaged 335 ± 17 (± SD) beats/min and 332 ± 9 (± SD) beats/min in the CONTROL and TREATMENT groups, respectively. Nighttime vs. daytime differences in mean arterial pressure and heart rate prior to commencing treatment are statistically significant at a *p* < 0.05.

Over the 2-week time span in which TGR(hAGT)L1623 rats were medicated with valsartan, the rats' systolic and diastolic blood pressures are significantly lower than those found in vehicle-treated animals (Figure 1). At the completion of a 2-week treatment period *daytime* arterial pressure averaged 148 ± 7/103 ± 4 (± SD) mm Hg in the control group and 130 ± 8/87 ± 4 (± SD) mm Hg in transgenic rats medicated with valsartan (*p* < 0.01). Administration of valsartan had no effect on heart rate either in terms of the average 24 h values or the presence of circadian rhythm differences in *daytime* and *nighttime* values (Figure 1).

**Figure 2:**
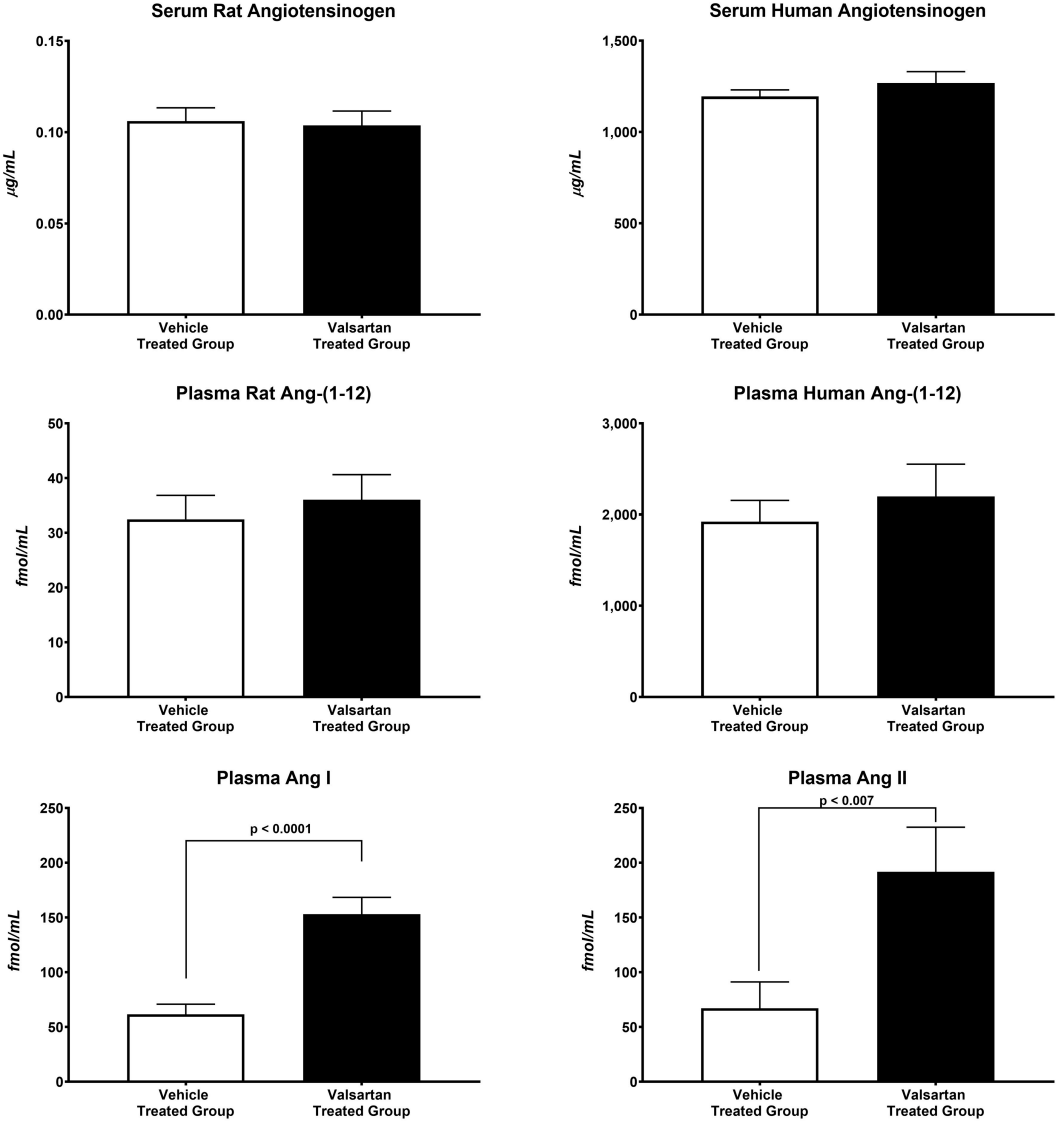
Blockade of AT_1_-R receptors results in statistically significant increases in plasma Ang I and Ang II concentrations. Values are means ± SE of plasma concentrations of rat and human Ang-(1-12) concentrations and correlative measures of plasma Ang I and Ang II as measured by RIA in 11 valsartan-treated and 11 vehicle-treated TGR(hAGT)L1623 adult male rats.

The antihypertensive response induced by blockade of AT_1_ receptors caused greater than a 10-fold increase in plasma renin concentration (7.48 ± 1.37 ng/mL/h in vehicle-treated rats vs. 84.11 ± 9.59 ng/mL/h in valsartan-treated rats; *p* < 0.0001) and no change in the serum concentrations of human and rat AGT concentrations (Figure 2). Baseline circulating levels of the human Ang-(1-12) sequence are ~57-fold higher than the rat sequence of plasma Ang-(1-12) concentrations (Figure 2). Blockade of AT_1_-R has no effect on plasma concentrations of rat and human Ang-(1-12). At the completion of the 14-day valsartan treatment regimen plasma Ang I and Ang II concentrations are markedly elevated compared with corresponding values in vehicle-treated transgenic rats (Figure 2).

**Figure 3:**
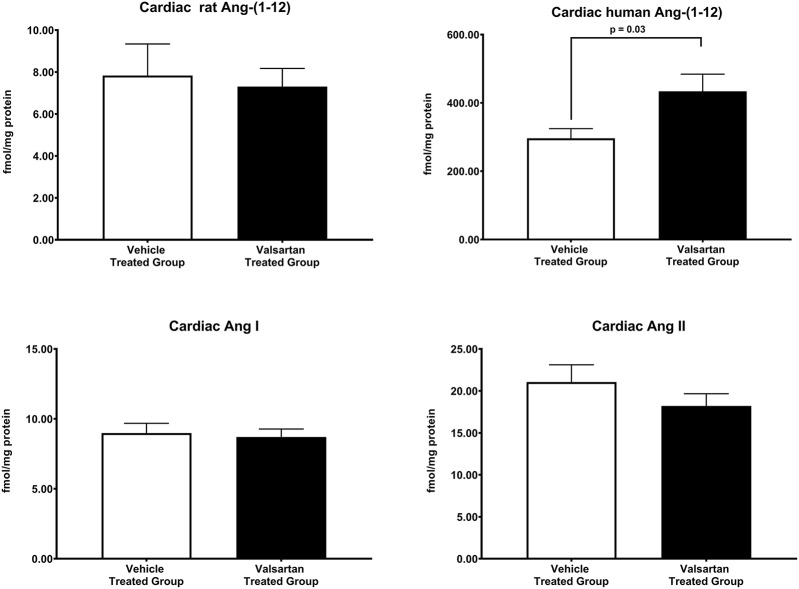
Normalization of blood pressure by valsartan-treatment produces a robust and selective increase in the human sequence of Ang-(1-12) in the left ventricular tissue of TGR(hAGT)L1623 rats. Values are means ± SE of left ventricular concentrations of rat and human Ang-(1-12) concentrations and correlative measures of cardiac Ang I and Ang II.

**Figure 4:**
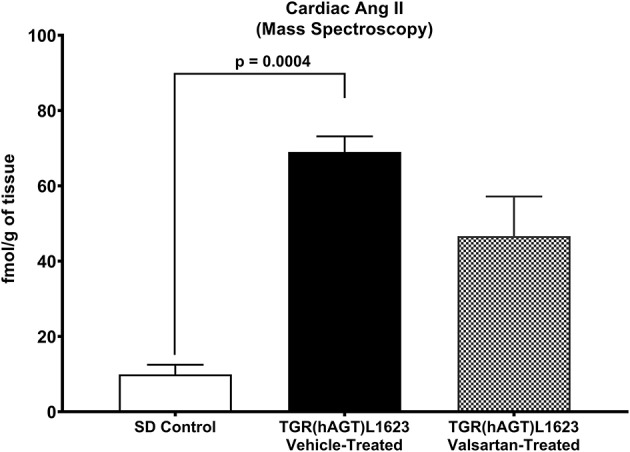
Comparative concentrations of left ventricular content of Ang II as determined by Mass Spectroscopy in normal Sprague Dawley (SD) rats and transgenic rats expressing the human angiotensinogen gene randomized to either treatment with vehicle or the AT_1_-R receptor antagonist valsartan. Values are means ± SE; *n* = 4 for each group.

**Figure 5:**
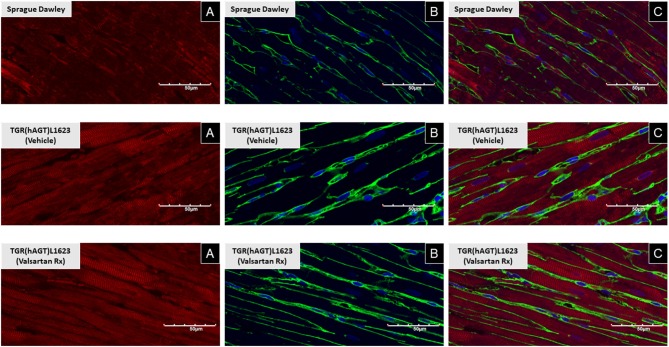
Immunofluorescence of chymase from left ventricular sections of a representative Sprague Dawley rat, a TGR(hAGT)L1623 rat treated with vehicle, and a TGR(hAGT)L1623 rat 2 weeks after initiating treatment with valsartan. Red chymase **(A)**, green/yellow wheat germ protein **(B)**, and blue DAPI **(C)**.

Figure 3 illustrates the grouped averages in the concentrations of Ang-(1-12), Ang I and Ang II measured in the left ventricle of transgenic TGR(hAGT)L1623 rats given the vehicle or valsartan. Here again, cardiac hAng-(1-12) content is much higher than rat Ang-(1-12) sequence (Figure 3). The antihypertensive treatment induces a 46% rise in cardiac hAng-(1-12) content (*p* < 0.03) and no changes in cardiac tissue levels of rAng-(1-12), Ang I and Ang II (Figure 3). Mass spectrometry analysis of angiotensins in left ventricular tissue obtained from hearts of 4 SD rats and 8 TGR(hAGT)L1623 heart [vehicle, *n* = 4; valsartan-treated, *n* = 4] confirmed the results obtained in the past study regarding a higher Ang II content in the heart of TGR(hAGT)L1623 hypertensive rats as measured by RIA (9). In agreement with the RIA measurements, a trend for reduced Ang II content following valsartan-treatment did not reach statistical significance (Figure 4). Cardiac concentrations for Ang-(1-9), Ang-(1-7), Ang-(1-5), Ang-(2-10), Ang-(2-7) Ang-(3-7), and Ang-(3-8) by LC/MS-MS were below the detectable level of the assay in SD rats and TGR(hAGT)L1623 rats medicated with either vehicle or valsartan (data not shown). Human AGT protein from the left ventricle of vehicle and valsartan treated rats averaged [1.22 ± 0.09 units/total protein and 1.19 ± 0.05 units/total protein (*p* > 0.05)], respectively. Cardiac chymase protein averaged 1.30 ± 0.05 units/total protein in vehicle-treated rats and 1.42 ± 0.11 units/total protein (*p* > 0.05) in those medicated with valsartan. Figure 5 documents increased cardiac chymase immunofluorescence in TGR(hAGT)L1623 rats with or without valsartan treatment compared to SD controls.

Given our previous findings documenting chymase as the primary Ang II-forming enzyme from Ang-(1-12) (20), we further assayed whether blockade of the type 1 Ang II receptors altered the expression of chymase gene transcripts in TGR(hAGT)L1623 rats. In agreement with the profile found in SHR and WKY rats (19), mast cell proteases (MCP-1 and MCP-5 showed equivalent expression in the heart of TGR(hAGT)L1623 rats (MCP-1: 0.928 ± 0.070 mRNA/GAPDH; MCP-5: 0.910 ± 0.058 mRNA/GAPDH; *p* > 0.05). Treatment with vehicle or valsartan did not change the expression of these gene transcripts. Likewise, cardiac ACE and ACE2 mRNAs showed no significant changes in response to valsartan treatment (data not shown). Cardiac chymase and ACE2 enzymatic activities were 5- and 4-fold higher than ACE cardiac enzymatic activity (Table 2). Valsartan treatment had no effect on the enzymatic activity of these enzymes (Table 2).

**Table 2:** Enzymatic activity of cardiac tissue angiotensin enzymes.

**TGR(hAGT)L1623 rats**	**Cardiac ACE activity (fmol/mg/min)**	**Cardiac ACE2 activity (fmol/mg/min)**	**Cardiac chymase activity (fmol/mg/min)**
Vehicle-treated	3.12 ± 0.40	12.46 ± 1.00	18.29 ± 1.79
Valsartan-treated	3.22 ± 0.37	13.50 ± 0.77	17.94 ± 1.68

*Values are means ± SE of the mean*.

Table 3 documents highly statistical correlations among cardiac hAng-(1-12), mean arterial pressure (*r* = 0.973) and plasma rAng-(1-12) (0.640). Similar significant correlations were found for plasma hAng-(1-12) with plasma Ang I (0.706) and cardiac rAng-(1-12) with cardiac ACE activity (−0.715). Cardiac chymase activity correlated with cardiac ACE2 activity (Table 3).

**Table 3:** Relationships among angiotensin-(1-12) and other angiotensin tissue determinants of angiotensin II activity in the vehicle treated group of TGR(hAGT)L1623 rats.

**Independent variable**	**Dependent variable**	**Correlation coefficient**	***P*-value**	**95% CI of slope**
Mean Arterial Pressure, (mm Hg)	Cardiac hAng-(1-12), (fmol/mg)	0.973	0.0055	0.52 to 1.34
Plasma rAng-(1-12), (fmol/mL)	Cardiac hAng-(1-12), (fmol/mg protein)	0.640	0.0339	0.03 to 0.54
	Cardiac ACE activity, (fmol/mg/min)	0.683	0.0206	−0.11 to −0.01
Plasma hAng-(1-12), (fmol/mL)	Plasma Ang I, (fmol/mL)	0.706	0.0152	0.01 to 0.03
Cardiac rAng-(1-12), (fmol/mg protein)	Cardiac ACE2 activity, (fmol/mg/min)	−0.715	0.0134	−0.82 to −0.12
Cardiac chymase activity, (fmol/mg/min)	Cardiac ACE2 activity, (fmol/mg/min)	0.753	0.0075	0.14 to 0.70

*CI, confidence interval*.

## Discussion

The creation of a rat expressing the human AGT gene in its genome allows for exploring non-renin mechanisms of excess Ang II activity as rat renin shows no catalytic activity for the human AGT protein (2). In the further characterization of this humanized hypertension model (9), we now show: (a) the presence of the human amino acid sequence of Ang-(1-12) in the blood and heart tissue of TGR(hAGT)L1623 transgenic rats; (b) validate our original demonstration of high cardiac Ang II concentrations in the heart of TGR(hAGT)L1623 hypertensive rats by MAS-spectroscopy; (c) demonstrate that blood pressure normalization with an AT_1_-R antagonist augments cardiac content of human Ang-(1-12) without concomitant changes in cardiac Ang I and Ang II concentrations; (d) describe for the first time cardiac expression of MCP-1 and MCP-5 chymase isoforms in rats expressing the human AGT gene; (e) document by confocal microscopy the presence of chymase immunofluorescence within cardiac myocytes; (f) quantitate a 4- to 6-fold higher ACE2 and chymase enzymatic activities compared to ACE activity in the hearts of TGR(hAGT)L1623 rats; (g) show that circulating hAng-(1-12) concentrations in these TGR(hAGT)L1623 rats correlates with mean arterial pressure (*r* = 0.973) and plasma Ang I concentrations (*r* = 0.706) and (h) report that a fortnight treatment with valsartan neither alters the human expression of AGT in either the blood or the heart tissue of these hypertensive rats. The collective characterization of the hemodynamic, biochemical, and rat and human expressions of renin angiotensin system components in the blood and cardiac tissue of rats expressing the human AGT gene offers new insights into the biological importance of alternate Ang II generating pathways whereby shorter sequences of the AGT substrate lead to chronic hypertension and cardiac hypertrophy through non-canonical renin angiotensin biotransformation pathways. While the identity of the enzyme cleaving the human AGT sequence in these transgenic rats remains to be fully identified, and it was not the object of this investigation, we showed previously that kallikrein or a kallikrein-like enzyme does cleave Ang-(1-12) from AGT (21, 22). The demonstration of higher cardiac hAng-(1-12) values during valsartan treatment suggest a potential for increased intracellular incorporation of Ang-(1-12) from the circulation or the interstitial spaces as demonstrated by us in neonatal cardiac myocytes (23) or alternatively, increased expression or activity of an AGT-cleaving protease. Further work will be necessary to explain the factor (s) accounting for the selective increase in cardiac hAng-(1-12) content following blockade of AT_1_-R.

Experimental models for the study of primary hypertension have yielded important information as to the diverse nature of the blood pressure regulatory mechanisms that contribute to blood pressure elevation (24). Too often, however, these studies highlight an overarching acceptance of renin as the primary pathway initiating the biochemical cascade leading to chronic Ang II pathological actions. While the humanization of rats with both the AGT and renin genes (25) reiterated the importance of renin mechanisms to chronic disordered blood pressure regulation, these studies did not exclude whether similar activation of hypertensive mechanisms may be initiated by increased expression of AGT alone. Global deficiency or reduction of AGT gene expression impairs survival and is associated with marked alterations in systemic and renal homeostasis (1). Likewise, AGT overexpression is associated with hypertension and renal tubular damage (26, 27). While the studies in mice implicate AGT expression as a source for altered structural and functional regulation of homeostatic mechanisms, no studies have addressed the pathogenetic role of Ang II-derived hypertensive actions in conditions in which renin does not initiate the substrate's hydrolysis.

While tissue Ang II synthesis is now generally accepted, a vigorous debate persists regarding the site within the tissue at which the peptide is formed (28–30). The original demonstration of a 4-fold increase in Ang II concentrations in the heart of TGR(hAGT)L1623 rats (9) is confirmed in the new data reported here. Cardiac Ang I and Ang II concentrations in vehicle treated transgenic rats are several orders of magnitude higher than those reported in normal and genetically hypertensive rats (31–35). The co-assessment of plasma and cardiac tissue concentrations of rAng-(1-12) and hAng-(1-12) now demonstrates that transcription of the human AGT into Ang-(1-12) drives the hypertension and augmented concentrations of Ang II in the heart of TGR(hAGT)L1623 rats. This interpretation is based on a <60-fold difference in plasma levels of rat and human Ang-(1-12) concentrations and an even higher difference between cardiac levels of rat and human Ang-(1-12). While this study does not directly answer the question of whether cardiac Ang II was formed intracellularly, increases in cardiac hAng-(1-12) content and chymase immuno-reactive fluorescence are in keeping with the idea that the elevated Ang II demonstrated in the heart of these transgenic rats is from intracellular formation. Previous studies from this laboratory did document increased Ang-(1-12) uptake in neonatal cardiomyocytes from SHR (23) and internalization of mast cell released chymase in isolated adult cardiac fibroblasts through a dynamin-dependent mechanism (36).

The hemodynamic response to a 2-week treatment with valsartan is in keeping with past studies reporting a significant antihypertensive effect of the drug at the doses employed here (14). We now show that blockade of AT_1_-R triggers an increase in plasma Ang I and Ang II concentrations in the absence of parallel elevations in rat and human levels of circulating Ang-(1-12). The absence of changes in circulating Ang-(1-12) may be interpreted to be related to the observation that rat renin which was significantly elevated in treated rats does not catalyze rat or human Ang-(1-12) (8). Therefore, the increases in circulating Ang I and Ang II during valsartan treatment originate from rat renin production of rat AGT-derived Ang I, blockade of Ang II receptor internalization by valsartan or both.

A differential response from valsartan's systemic effects was documented in the heart of TGR(hAGT)L1623 rats. Treatment with the AT_1_-R antagonist was associated with a significant increase in the concentration of hAng-(1-12) while rAng-(1-12), Ang I, and Ang II did not change. Numerous studies demonstrate an intracellular Ang II expression of local origin [see (37) for review] and consensus is emerging regarding the existence of an intracellular cardiac RAS that functions independently from the circulating RAS (28, 38, 39). Independent assessment by Mass-Spectroscopy confirmed the data obtained by RIA measurements. With both methods, a trend for reduced cardiac Ang II content did not reach statistical significance. These data are in keeping with similar findings from previous studies where blockade of AT_1_-R failed to alter cardiac Ang II content (32, 33, 35, 40). As reviewed recently (41–43), clinical trials of RAS inhibitors failed to show a large benefit in cardiac outcomes beyond what can be attributed to the blood pressure lowering effect of these drugs. This discrepancy, as noted by Kumar et al. (38), may reflect the inability of RAS blockers to gain access to the intracellular compartments at which Ang II is generated.

Ang-(1-12) processing into biologically active peptides is mediated by ACE in the circulation (44) and chymase in cardiac and renal tissues (10, 20, 45). Corresponding to the heightened gene expression levels of MCP-1 and MCP-5, but not the other MCPs, in hearts of hypertensive and normotensive female rats (19), we show that these two cardiac chymase isoforms are also expressed in the heart of TGR(hAGT)L1623 rats. In keeping with previous studies, chymase has been visualized in human (46), dog (47, 48), and enlarged rat myocytes from volume overload due to an aorto-caval fistula (21, 49). Confocal microscopy using an antibody directed to the human chymase (CMA-1) demonstrated the presence of strong chymase immunofluorescence in rats expressing the human AGT gene. The enrichment of chymase in the heart of TGR(hAGT)L1623 rats coincides with the concurrent finding of high chymase activity using Ang-(1-12) as a substrate (20). Taken together with the heightened Ang II in hearts of transgenic when compared to control SDs, these findings substantiate the view that Ang forming-chymase is central to the conversion of Ang-(1-12) to Ang II.

In summary, rats expressing the human AGT gene are hypertensive and are characterized by a RAS profile with higher circulating and cardiac levels of human Ang-(1-12). Treatment with valsartan results in augmented concentrations of circulating Ang I and Ang II as well as increased cardiac human but not rat Ang-(1-12). Both here and in the previously published study (9) the hypertension and cardiac dysfunction characterized in a humanized model of hypertension is validated through the confirmation that expression of the human AGT gene does not alter circadian blood pressure mechanisms as the more pronounced hypertension and tachycardia, observed during the night hours, reflects the augmented locomotor activity of nocturnal animals. We also confirmed by both RIA and MASS-Spectroscopy the existence of high Ang II concentrations in the heart of TGR(hAGT)L1623 rats and now report the presence of elevated cardiac myocyte chymase expression. Moreover, we show that cardiac chymase enzymatic activity is several orders of magnitude greater than ACE or ACE activities.

Given the existence of a robust vast literature dealing with chymase contribution to the pathogenesis of diseases of the heart and blood vessels (50, 51), controversy as to its role in mediating Ang II-derived pathology is perplexing. Limitations in terms of experimental design, the presence of high concentrations of endogenous protease inhibitors in interstitial fluid and dismissing the fact that chymase can be sourced from either degranulation of mast cells or gene translation in myocytes, fibroblast and endothelial cells has contributed to discard this noncanonical pathway of Ang II production as a critical mechanism contributing to adverse cardiac remodeling. The void has been further aggravated by a slow pace in the development of specific chymase inhibitors, a fact that may be finally circumvented by the promising findings of recent experimental studies (45) and a published clinical trial (52, 53).

## Data Availability Statement

The datasets generated for this study are available on request to the corresponding author.

## Ethics Statement

The animal study was reviewed and approved by the Animal Care and Utilization Committee, Wake Forest School of Medicine.

## Author Contributions

CF and JVa designed the study. CF wrote the manuscript, analyzed the data, and prepared the figures. SA and KW performed the enzymatic assays, analyzed the data and contribute to manuscript writing. HW performed the gene transcript studies and analyzed the data. TY and DR performed the immunohistochemistry studies and contributed to data analysis. JVo performed echocardiographic studies. LG analyzed data, and assisted in manuscript writing. JC, CC, and LD assisted in interpretation of results and manuscript revision. JVo and KW performed hemodynamic, echocardiographic, and biochemical studies. JC and LD assisted in the writing of the manuscript.

### Conflict of Interest

The authors declare that the research was conducted in the absence of any commercial or financial relationships that could be construed as a potential conflict of interest.
